# Motor disturbances and thalamic electrical power of frequency bands' improve by grape seed extract in animal model of Parkinson's disease

**Published:** 2012

**Authors:** Alireza Sarkaki, Zainab Eidypour, Freshteh Motamedi, keivan keramati, Yaghoub Farbood

**Affiliations:** 1 *Physiology Research Center (PRC), Medicinal Plant Research Center, Ahvaz Jundishpur University of Medical Sciences, Ahvaz, I.R. Iran*; 2 *Department of Biology, Faculty of Sciences, Damghan Islamic Azad University, Damghan, I.R. Iran. *; 3 *Iranian Neurosciences Research Network and Neurosciences Research Center, Shahid Beheshti University of Medical Sciences, Tehran, I.R. Iran*; 4 *Faculty of veterinary Medicine, Semnan University, Semnan, I.R. Iran*; 5 *Department of physiology, Medicine Faculty, Physiology Research Center (PRC), Ahvaz Jundishpur University of Medical Sciences, Ahvaz, I.R. Iran*

**Keywords:** Grape seed extract, Motor disorders, Thalamic electrical power, 6-hydroxydopamine, Parkinson's disease, Rat

## Abstract

**Objective:** Previous studies showed that grape seed extract (GSE) is an excellent natural substance with potent antioxidant effect and free radical scavenger. This study aimed to evaluate the effect of GSE on motor dysfunctions and thalamic local Electroencephalography (EEG) frequency bands' powers in rats with Parkinson's disease (PD).

**Materials and Methods:** In this study 8 µg 6-hydroxydopamine (6-OHDA) dissolved in 2 µl normal saline containing 0.01% ascorbic acid was infused into right medial forebrain bundle (MFB) to make an animal model of PD. Rats with PD received four weeks GSE (100 mg/kg, p.o.) after apomorphine-induced rotation test. Spontaneous motor tests and also thalamic ventroanterior nucleus (AV) local EEG recording were done in freely moving rats in all groups.

**Results:** Chronic treatment of PD rats with GSE could influence potentially frequency bands' powers of thalamic VA and improve post-lesion motor dysfunctions significantly (p<0.05 and p<0.01, respectively).

**Conclusion:** Our findings suggest that GSE modulates the CNS function and has beneficial effects on the direct and indirect striato-thalamo-cortical pathways in PD. GSE acts as a new and potent natural free radical scavenger which removes oxidants produced by neurotoxin 6-OHDA in brain. Therefore, it reinforces electrical power of remained thalamic VA neurons and thereby improves post-lesion motor disorders.

## Introduction

The unilateral injection of 6-hydroxydopamine (6-OHDA) in substantia nigra pars compacta (SNpc) or medial forebrain bundle (MFB) in rats is frequently used to make an animal model of Parkinson's disease (PD). MFB lesion model mimics an early stage of PD (Kim et al. 2010 ▶; Sun et al. 2010 ▶). PD is characterized by the bilateral degeneration of the midbrain dopamine-containing neurons with the most severe lesion in the posterior-lateral part of the substantia nigra pars compacta (SNpc). In humans, such lesions lead to specific motor abnormalities (i.e., akinesia, rigidity, and tremor) that are greatly improved by drug treatment (Paille et al. 2007 ▶). The 6-OHDA model of Parkinson's disease in the rat represents a fundamental tool for investigating the pathophysiology of dopamine denervation (Fulceri et al. 2006 ▶). Since 6-OHDA injections into the MFB and the striatum (STR) result in complete and partial SNpc lesions, respectively, it is believed that communication links exist between neurons, along neuronal pathways that transmit activating signals in response to neuronal damage (Henning et al. 2008 ▶). 

The aerial parts of Vitis vinifera (common grape or European grape) have been widely used to treat a variety of common and stress-related disorders (Sreemantula et al. 2005 ▶). Grape seed extract (GSE) is a new natural free radical scavenger, a potent antioxidant and neuroprotective. It lowers body temperature, but reduces brain injury even when body temperature is normal (Cetin et al. 2008a ▶; Cetin et al. 2008b ▶). GSE reduces neurofunctional abnormalities caused by the hypoxia-ischemia (HI) (Feng et al. 2007 ▶). Dietary supplements such as GSE enriched in proanthocyanidins (PA) (oligomeric polyphenols) have been suggested to have multiple health benefits, due to antioxidant and other beneficial activities of the PA. Proanthocyanidins are potent natural antioxidants which belong to a class of polyphenols and are prepared from grape seeds (Devi et al. 2006 ▶; Karaaslan et al. 2010 ▶). Grape seed proanthocyanidins extract (GSPE), whose principal ingredient is proanthocyanidins, shows many activities such as cholesterol lowering, antioxidant, anti-tumor, cardioprotective, and protection against ultraviolet rays (Gunjima et al. 2004 ▶). GSE is a commonly available dietary supplement taken for the anti-oxidant activity that's attributed to its proanthocyanidin content (Kim et al. 2006 ▶). 

Normal nigrostriatal system affects the thalamic and ultimately cortical neurons activity via direct and indirect GABAergic projections to globus pallidus/substantia nigra nuclei as well as glutamatergic inputs from subthalamic nucleus. Lesions to the SNpc or MFB by neurotoxins cause changes in striatal D1 and D2 receptors activity and disrupt brain function which play a major role in the impaired movement (bradykinesia) associated with reduced dopaminergic function (London et al. 2010 ▶). The neurotoxin-administered animal subject (PD induced by 6-OHDA) exhibited average forelimb step distance (stride length) that was lower than control (82.58%) and correlated with the striatal DA levels, and this correlated well with the degree of striatal dopamine (DA) depletion (Kim et al. 2010 ▶; Sameri et al. 2011 ▶). Since the brain is vulnerable to age-related oxidative damage during neurodegenerative disorders such as PD, Alzheimer’s disease (AD), and other insults including inflammation. So, according to the above-mentioned knowledge, we investigated the effect of chronic administration of GSE on motor dysfunction, thalamic ventroanterior nucleus local EEG, and its frequency bands' power two weeks after PD induction in rats. 

## Materials and Methods

In this study, 38 healthy middle-aged male Wistar rats (24–30 months; weighing 350±30 g) were obtained from Ahvaz Jundishapur University of Medical Sciences (AJUMS) laboratory animal center. Animals were housed individually in standard cages under controlled conditions; room temperature (20±2 °C), humidity (35-50%) and 12 h light/dark cycle (light on at 07:00 am). All experiments were carried out during the light phase of the cycle (08:00 am to 06:00 pm). Access to food and water were ad libitum except during the tests. Animal handling and experimental procedures were performed under observance of the University and Institutional legislation, controlled by the Local Ethics Committee for the Purpose of Control and Supervision of Experiments on Laboratory Animals. All efforts were made to minimize animal suffering and reduce the number of animals used. Prior to the onset of behavioral testing, all animals were gently handled for 5 days (5 minute daily). The animals were divided randomly into five groups of 7-8 each: 1) control, intact rats, 2) sham operated (Sham-PD), received 2 µl normal saline containing 0.01% ascorbic acid into right medial forebrain bundle (MFB), 3) lesioned (PD), received 8 µg/2µl 6-hydroxydopamine dissolved in normal saline with 0.01% ascorbic acid into right MFB, 4) PD-GSE, MFB-lesioned rats which received grape seed hydro alcoholic extract (GSE, 100 mg/kg, p.o.) for 28 days, and 5) PD-Veh, lesioned rats received same volume of vehicle (normal saline with 0.01% ascorbic acid). A single dose of GSE was selected as effective dose based on the dose response study carried out in our laboratory previously (Badavi et al. 2008 ▶; Farbood et al. 2009 ▶; Sarkaki et al. 2007 ▶).

**Animal model of PD**

**Figure 1:**
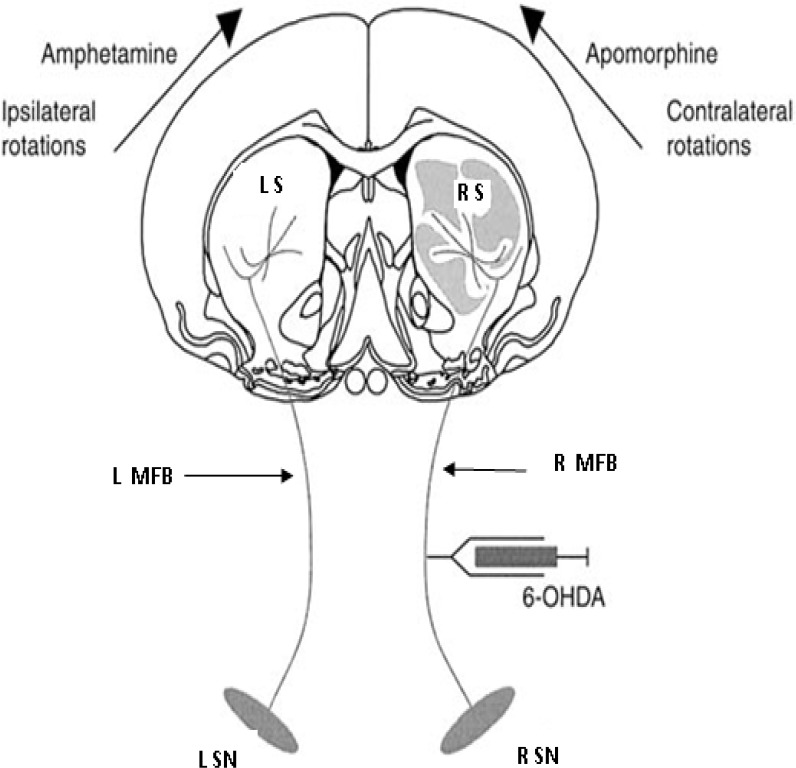
Diagram of the nigrostriatal pathway and rotational responses produced by apomorphine (contralateral rotations). Shaded areas in the right striatum indicate the loss of DA due to a MFB injection of 6-OHDA. Abbreviations: RS: right striatum, LS: left striatum, R MFB: right medial forebrain bundle, L MFB: left medial forebrain bundle, 6-OHDA: 6-hydroxydopamine, R SN: right substantia nigra, and L SN: left substantia nigra. Figure is adapted with some modifications    (Deumens et al. 2002b ▶)

Medial forebrain bundle (MFB) in the right brain hemisphere was lesioned using the Tadaiesky's (2008) ▶ method with some modifications (Tadaiesky et al. 2008 ▶). Briefly, stereotaxic surgery was performed under ketamine/xylazine (100/10 mg/kg, i.p.) anesthesia. Surgeries were done using the coordinates in Paxinos and Watson atlas: AP: -4.4 mm, ML: -1.2 mm, and DV:-8.2 mm from bregma and skull surface (Paxinos and Watson 2007 ▶). Eight µg/2µl 6-hydroxydopamine HBr (Sigma, MO) were dissolved in normal saline with 0.01% ascorbic acid and was infused into right MFB using a 10 µl Hamilton syringe with a 26-gauge needle connected to a 30-gauge cannula. Following injection, the cannula was left in place for 5 minute before being retracted to allow complete diffusion of the drug. All animals were treated with (i.p.) injection of 25 mg/kg desipramine (Exeir Pharmacy Co., Iran) 30 minute before surgery, in order to protect noradrenergic terminals depletion by 6-hydroxydopamine (6-OHDA). Sham-operated rats followed the same protocol except for the fact that vehicle was injected instead of 6-OHDA (Figure 1). Afterward, a coated stainless steel bipolar metal wire electrode (stainless steel Teflon, 0.005" bare, 0.008" coated, A-M systems, Inc. WA) was implanted in the right thalamic ventroanterior (VA) nucleus at AP: -2.16 mm (from bregma), ML: -1.8 mm, DV: -6.4 mm (from skull surface), (Paxinos and Watson 2007) ▶. All implants were fixed to the skull by acrylic cement and two glass anchor small screws. The local EEG recording and motor tests were done 14 days after recovery from the surgery.

**Grape seed extract preparation**

Large clusters of red grapes were purchased from Qazvin province gardens (northwest of Iran), as Vitis vinifera (Linn). Seeds were removed from the grapes, air dried (in shade) for one week and milled to fine powder (a particle size of <0.4 mm). The powder was macerated in 70% ethanol (25% w/v) for 72 h at room temperature and was stirred three times a day. The ethanol extract evaporated and the extract was obtained as a lyophilized powder (yield: 25-30%). GSE extract was dissolved in normal saline and animals in treated groups received a daily dosage of 100 mg/kg by oral gavage (p.o.) for 4 weeks.

**Drug-induced rotational behavior**

Motor asymmetry following unilateral lesion of the MFB was assessed by apomorphine-induced rotational behavior. The rats were injected subcutaneously with 0.5 mg/kg apomorphine hydrochloride (Sigma, USA) dissolved in normal saline containing 0.01% ascorbic acid to confirm the dopamine depletion in nigrostriatal system. Total turns contralateral to the lesion side were counted over a period of 15 minute. All groups were tested for rotational behavior 14 days after the lesion (Rizelio et al. 2010 ▶) and just before every treatment (Figure 1). 

**Local EEG recording**

Electrical field potentials (local EEG) from the right thalamic VA nucleus of freely moving rats were fed to a ML135 bio-amplifier (AD Instruments, 4-Channels Power Lab, LabChart software version 7, Australia) with 1 mV amplification, sample recording 400 Hz, and 0.3–70 Hz band pass filtration for 5 minute. The crude EEG and its gamma, alpha, beta, theta, and delta bands amplitude changes during a period of 5 seconds were compared in all groups. Electrical power of frequency bands were measured as µV^2^/Hz (Sameri et al. 2011 ▶; Sarkaki et al. 2008 ▶).

**Catalepsy tests**

The catalepsy was assessed by placing one forepaw on a horizontal bar 9 cm above the surface, and another forepaw on a podium (3 cm high). The latency to initiate the movement was used as a measure of catalepsy. Potency of a tested substance to decrease the latency in the catalepsy test was considered indicative of its potential anti-akinetic effect. Afterward, muscle stiffness (rigidity) was tested. The scoring adopted was based on a three stage model as follows: Stage 1: when the rat was placed on a flat table, if it showed normal movement, the score allocated was 0, if the rat did not move, but on gentle touch it showed movement, the allocated score was 0.5, Stage 2: one of the rats' forepaws was placed on a 3 cm high wooden podium block, if the rat did not replace its position within 10 s, it received a score of 0.5. Similarly, the second forepaw was placed on the wooden block and scored similarly, and Stage 3: one of the forepaws was placed on a 9 cm high wooden block and another paw left hanging. A positive sign for full rigidity was gauged by the failure of the animal to correct the imposed position within 10 s and was given a score of 1. A similar procedure was used with another forepaw. Therefore, if a rat was in full rigidity (muscle stiffness), a total cumulative score of 3.5 was assigned (Dekundy et al. 2006 ▶; Sameri et al. 2011 ▶; Sarkaki et al. 2008 ▶).

**Stride length test**

Stride length was tested in intact, PD, and PD groups after chronic treatment with GSE (100 mg/kg, p.o, once daily for 28 days) or vehicle. The apparatus was composed of a woody box (20×17×10 cm), in which a runway (4.5 cm wide, 42 cm long with borders of 10 cm height) was arranged to lead out into dark wooden box. Stride lengths were measured by wetting animals' forepaws with commercially available pencil blue or red inks and letting them trot on a strip of paper (4.5 cm wide, 40 cm long) down the brightly lit runway towards the dark goal box. The forelimb stride lengths were measured for all animals (Control, PD, Sham-PD, and PD-GSE or PD-Veh). Stride lengths were measured manually as the distance between two forepaw prints. The three longest stride lengths (corresponding to maximal velocity) were measured from each run. Paw prints made at the beginning (7 cm) and the end (7 cm) of the run were excluded because of velocity changes. Runs in which the rats made stops or obvious decelerations observed by the experimenter were excluded from the analysis (Fernagut et al. 2002 ▶; Sameri et al. 2011 ▶). 

**Motor coordination test**

The rotarod test was performed two weeks after lesion or after 28 days of chronic treatment with GSE or vehicle in lesioned groups. The rotarod test served the purpose of detecting potential deleterious effects of the compounds studied on the rats' motor performance and coordination. The animals were placed on a testing rod of the accelerating rotarod apparatus (Borj Sanaat Co., Iran) at an initial speed of five rotations per minute (5 rpm) for the first 5 minutes, then, speed was increased gradually to 40 rpm over 10 minutes. The animals were pre-trained to reach a stable performance in this test. The training consisted of three sessions on three consecutive days, and each session included three separate testing trials. In each session test, animals were placed on the rod at 45–60 minutes intervals before and after chronic treatment with GSE. The rotarod performance was expressed as time spent on the rod at accelerating velocities expressed in seconds (Dekundy et al. 2006 ▶). 

**Statistical analyses**

Data were expressed as mean±SEM of values for motor activity tests, local EEG of the thalamic VA nucleus, and its frequency bands' electrical power (µV^2^/Hz). Statistical analysis was performed by Kruskal-Wallis followed by post-hoc median test for rigidity and by one-way ANOVA followed by LSD post-hoc test for other data. A p-value less than 0.05 were assumed to denote a significant difference and levels of significance are indicated by symbols: *p<0.05, **p<0.01, ***p<0.001.

**Drugs**

Apomorphine and 6-OHDA were purchased from Sigma Chemical Co., USA and Ketamine HCl and xylazine from Alfasan, Woerden, Holland. Desipramine was obtained from Exeir Pharmacy Co., Iran.

## Results

**Motor tests**

**Figure2:**
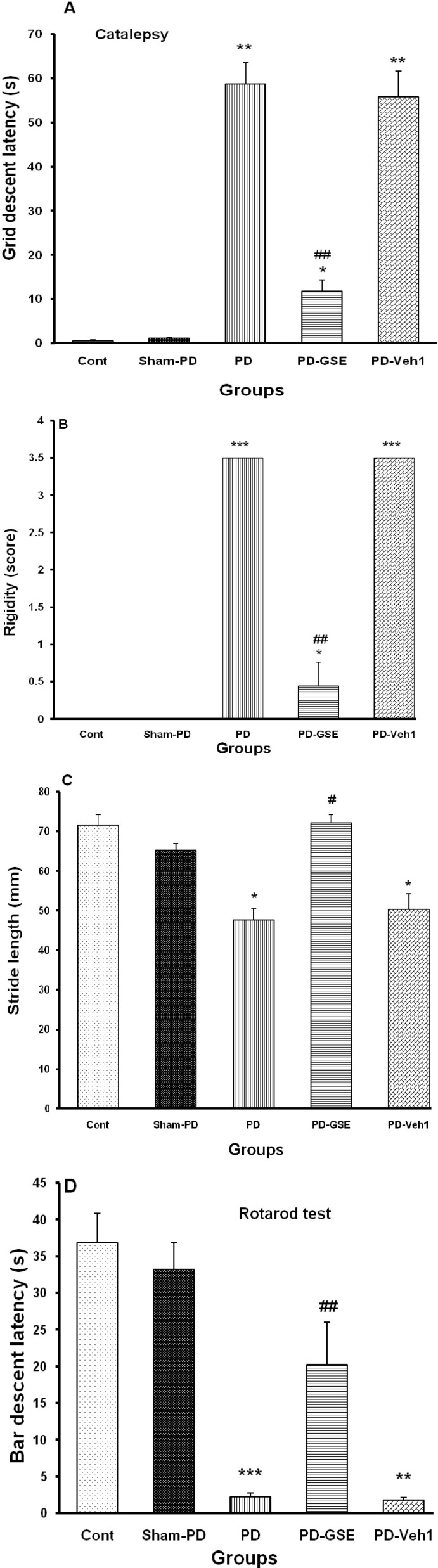
Motor function tests.** A)** Grid descent latency (s) in PD increased significantly (**p<0.01 and *p<0.05 for PD, PD-GSE and PD-Veh. vs. control and sham-PD. Chronic treatment of PD with GSE decreased it significantly (**##**p<0.01 for PD-GSE vs. PD or PD-Veh), **B)** Rigidity or muscle stiffness score in PD increased significantly (***p<0.001 and *p<0.05 for PD, PD-GSE and PD-Veh. vs. control and sham-PD.for PD and PD-Veh. vs. control and sham-PD). Chronic treatment of PD with GSE reduced score of rigidity significantly (**##**p<0.01 for PD-GSE vs. PD or PD-Veh, and *p<0.05 for PD-GSE vs. control and Sham-PD), **C)** Stride length (mm) in PD decreased significantly (*p<0.01 for PD and PD-Veh vs. control and sham-PD). Chronic treatment of PD with GSE increased reversed it significantly (#p<0.05 for PD-GSE vs. PD and PD-Veh), and **D)** Bar descent latency (s) as motor coordination decreased significantly in PD (***p<0.001 and **p<0.01 for PD and PD-Veh vs. control and sham-PD respectively). Chronic treatment of PD with GSE increased it latency significantly (**##**p<0.01 for PD-GSE vs. PD and PD-Veh). Kruskal-Wallis followed by median test for rigidity test and one-way ANOVA followed by LSD post-hoc test for other motor tests.

Data have shown that in PD group, the grid descent latency of forepaws on a 9 cm height bar, as a valuable parameter for bradykinesia (catalepsy), was increased significantly (p<0.01) when compared with control or Sham-PD groups, while chronic treatment with GSE reversed it significantly (p<0.01 for PD-GSE vs. PD and PD-Veh groups, Figure 2A). Muscle stiffness (rigidity) was increased significantly in PD (p<0.001 for PD vs. control or sham-PD groups), while chronic treatment with GSE decreased it significantly (p<0.01, Figure 2B). Results of forepaws walk length prints (by stride-length test) showed that walk length in PD was significantly lower than that in control or sham-PD groups (p<0.05). Chronic treatment of PD rats with GSE could increase walk length significantly (p<0.05 for PD-GSE vs. PD and PD-Veh, Figure 2C). Data obtained from all groups following motor balance test in rotarod showed that bar descent latency in PD group was decreased severely when compared with control or sham-PD groups (p<0.001), while chronic treatment with GSE could improve significantly disrupted motor balance induced by 6-OHDA lesion (p<0.01 for PD-GSE vs. PD and PD-Veh, Figure 2D).

**Electrophysiology of thalamic VA nucleus**

**Figure3:**
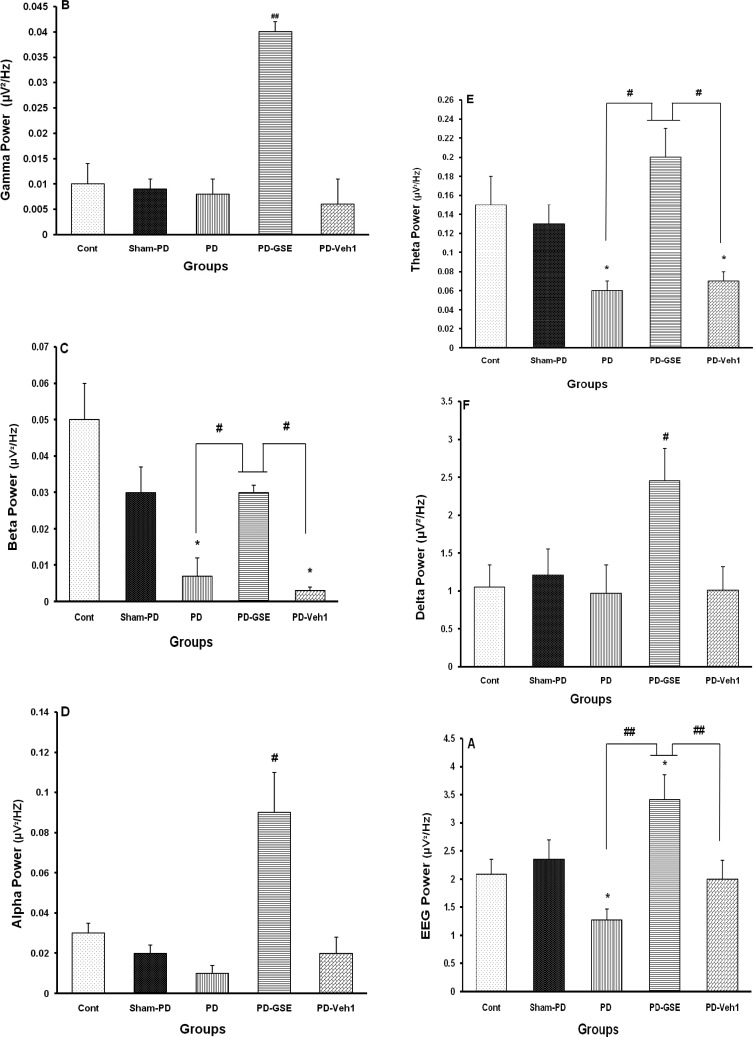
Thalamic VA local EEG and its frequency bands' powers (µV^2^/Hz). **A)** EEG electrical power was decreased in PD significantly (*p<0.05), and chronic treatment with GSE restored it (##p<0.01 for PD-GSE vs. PD and PD-Veh), **B)** Gamma wave power didn't change after MFB lesion while it was increased in PD after chronic treatment with GSE significantly (**##**p<0.01, PD-GSE vs. PD and control and Sham-PD groups), **C)** Beta wave power was decreased in PD significantly (*p<0.05 for PD and PD-Veh vs. control and Sham-PD groups). After chronic treatment of PD with GSE it was reversed to control level (#p<0.05 for PD-GSE vs. PD and PD-Veh), **D)** Alpha wave power didn't change after MFB lesion while it was increased after chronic treatment with GSE significantly (#p<0.05, PD-GSE vs. PD-Veh and other groups), **E)** Theta power was decreased in PD significantly (*p<0.05 for PD vs. control). After chronic treatment with GSE it was reversed (#p<0.05 for PD-GSE vs. PD-Veh), and **F)** Delta wave power didn't change after MFB lesion, it was increased after chronic treatment with GSE significantly (#p<0.05, PD-GSE vs. other groups). One-way ANOVA followed by LSD post-hoc test, n=8).

The electrical power of recorded thalamic VA local EEG were decreased significantly after lesioning the MFB by 6-OHDA (p<0.05 for PD vs. Control or Sham-PD groups). Chronic treatment of PD with GSE significantly increased EEG power (p<0.01 for PD-GSE vs. PD and PD-Veh). Therefore, GSE could restore thalamic local EEG power in PD and thereby improve its motor dysfunctions (Figure 3A). The electrical power of gamma band in recorded thalamic VA local EEG did not change after damaging the MFB by 6-OHDA when compared with control or sham operated (sham-PD) groups, while chronic treatment with GSE significantly increased it when compared with all other groups (p<0.01). Therefore, GSE could increase thalamic gamma band power in PD and thereby affect its awareness and motor functions (Figure 3B). The electrical power of beta band of thalamic VA local EEG was decreased significantly in PD (p<0.05 for PD vs. control or sham-PD groups). Chronic treatment of PD with GSE significantly increased beta power (p<0.05 for PD-GSE vs. PD and PD-Veh groups). Therefore, GSE could reverse thalamic beta power in PD and thereby affect its awareness and motor functions (Figure 3C).The electrical power of alpha band of thalamic VA local EEG did not change significantly after lesion the MFB by 6-OHDA when compared with control or sham operated (Sham-PD) groups, while chronic treatment of PD with GSE significantly increased it when compared with all other groups (p<0.05). Therefore, GSE could increase thalamic alpha band power in PD and thereby affect the motor functions (Figure 3D). The electrical power of theta band of thalamic VA local EEG was decreased significantly in PD (p<0.05 for PD vs. control or sham-PD groups). Chronic treatment with GSE increased it significantly (p<0.05 for PD-GSE vs. PD and PD-Veh groups). Therefore, GSE could reverse thalamic beta power in PD (Figure 3E). The electrical power of delta band of thalamic VA local EEG did not change after lesioning the MFB by 6-OHDA when compared with control or sham operated (sham-PD) groups, whiles chronic treatment of PD with GSE significantly increased it when compared with all other groups (*p<0.05, Figure 3F).

## Discussion

We found that chronic treatment of PD rats with GSE caused extensive improvement of the motor disorders as well as thalamic VA local EEG frequency bands' power, significantly. We used MFB lesion model because it was established as an animal model of PD using injection of 6-OHDA into the MFB and has advantages over other models. It has been shown that there is a similar tendency in the striatal DA transporter decrease both in MFB-lesioned rats and other rat models (striatal lesion, etc.) 4 weeks after lesion, however, an increase (up-regulation) of dopaminergic D2-like receptor in the MFB-lesioned rats has been shown, whereas a decrease (down-regulation) in the striatal-lesioned rats has been documented (Sun et al. 2010 ▶). The same finding was obtained by other investigators, too. Increasing evidences have shown loss of dopamine content in the striatum of rats and also some of principal neurotransmitters level in the brain (parts affecting motor and cognition) after MFB lesion and it is a model mimicking end-stage Parkinson's disease. However, the partial denervation renders is quite suitable for mimicking early stage of Parkinson's disease, so this is proper for testing possible neuroprotective and neurotrophic drugs (Yuan et al. 2005 ▶). We also began rotation test with apomorphine 14 days after lesioning. Only lesioned animals with beginning contralateral rotation 5-10 minutes after receiving apomorphine (about 100 rotations during 10 minute observation) were included in the study. 

Despite a 90% dopamine cell loss in the substantia nigra (SN) five weeks after MFB lesion, extracellular dopamine levels in the SN are kept at near-normal levels. However, the response to a pharmacological challenge is severely disrupted (Sarre et al. 2004 ▶). Destruction of the ipsilateral MFB using 6-OHDA abolished ipsiversive circling but enhanced contraversive circling produced by dopamine or apomorphine. Ipsiversive circling produced following intranigral injection of dopamine is dependent upon the integrity of ascending dopamine neurons. Contraversive rotation is independent of ascending dopamine pathways but is reliant upon afferent input to the substantia nigra from the striatum and/or globus pallidus (Kelly et al. 1984 ▶). Independent from the site of injection, the 6-OHDA-induced DA depletion appears to be a valuable model to investigate PD symptomatology and to gain more insight into the possible pathological mechanisms of this neurodegenerative disease (Deumens et al. 2002a ▶). As mentioned above, lesioning the MFB may impair the striatal dopamine (DA) content, DA receptors density, neurotransmitter levels in CNS, neural activity, neuronal plasticity in crucial brain areas, and increase brain free radicals followed by motor, cognition, and electrophysiological disorders. Dietary supplements such as grape seed extract (GSE) enriched in proanthocyanidin (PA) have been suggested to have multiple health benefits, due to antioxidant and other beneficial activities of the PA (Cetin et al. 2008b ▶; Chis et al. 2009 ▶). 

Studies showed that PA intake in moderately low quantity (oral administration) is effective in up-regulating the antioxidant defense mechanism by attenuating lipid peroxidation (LPO) and protein oxidation (PO) (Farbood et al. 2009 ▶; Sarkaki et al. 2007 ▶). The neuroprotective effects of grape seed proanthocyanidin (GSPE) on the cerebral cortex (CC), cerebellum (CB), and hippocampus (HC) in the adult rat brain were similar to an increased superoxide dismutase (SOD) activity in the PA-supplemented animals, with a substantial decrease in malondialdehyde (MDA) and protein carbonyl content (PCC) (Sangeetha et al. 2005 ▶). Therefore, these findings suggest that GSE is an effective anti-aging substance to prevent the oxidative stress. 

Parkinson's disease (PD) is closely associated with increased free radical production, which ultimately leads to devastation of normal cell function and membrane integrity (Sangeetha et al. 2005 ▶). GSE improves stress-induced cognition impairment in rats as dose dependent. The extract also produced significant inhibition of hydroxyl radicals in comparison with ascorbic acid in a dose-dependent manner (Sreemantula et al. 2005 ▶). Therefore, our findings with previous studies provide scientific support for the anti-stress (adaptogenic), antioxidant, and nootropic activities of V. vinifera seed extract and substantiate the traditional claims for the usage of grape and its seeds in stress-induced disorders. 

Disruption of motor cortex activity is hypothesized to play a major role in the slowed movement (bradykinesia) associated with reduced dopaminergic function. Bradykinesia is also associated with decreased intensity of bursting and amplitude of cross-correlation peaks at rest. 

In the current study, the results show for the first time that significant reductions can be detected in motor cortex activity at rest in animals with impaired ability to generate movements induced by reduced dopamine action and confirm that impaired movements are associated with reduced cortical activation (Parr-Brownlie and Hyland 2005 ▶). The mechanisms whereby thalamus regulates dopamine release may be exerted via a local circuit and/or a pre-synaptic mechanism in the region of dopamine terminals (Kilpatrick et al. 1986 ▶). 

Therefore, it seems that dopamine release in the caudate-putamen is sensitive to experimentally induced changes in neural activity and damages of its thalamic input. Thalamic VA local EEG power was affected in PD, so it can lead to motor disorders. Chronic treatment with GSE reversed impaired motor behaviors and thalamic EEG; especially its gamma and beta bands electrical powers induced by PD. 

Chronic treatment with GSE could reverse motor disorders and power of thalamic EEG frequency bands significantly in rats with PD. Our results suggest that reversion of thalamic electrical power to normal level requires consumption of some effective preparations and constituents of GSE such as gallic acid, GSPE, etc. as nutritional supplements.
